# Spectroscopic Investigations of a Vandalized Contemporary Acrylic Painting on Canvas Using Model Paintings and Chemometrics

**DOI:** 10.1002/cplu.202500129

**Published:** 2025-06-13

**Authors:** Jana Striova, Silvia Innocenti, Arianna Ingrassia, Moira Bertasa, Barbara Salvadori

**Affiliations:** ^1^ National Institute of Optics National Research Council Largo Enrico Fermi 6 50125 Firenze Italy; ^2^ Department of Ancient Sciences La Sapienza, University of Rome p. Aldo Moro 5 00186 Rome Italy; ^3^ Settore restauro Dipinti su tela e tavola Opificio delle Pietre Dure V.le Filippo Strozzi, 1 50129 Firenze Italy; ^4^ Conservation Collections Care and Collections Management Department Historic Royal Palaces Hampton Court Palace, East Molesey Surrey KT8 9AU UK; ^5^ Institute of Heritage Science National Research Council Via Madonna del Piano 10 Sesto Fiorentino (FI) Italy

**Keywords:** acrylic model samples, ageing, contemporary artwork, felt‐tip marker pen, vandalism

## Abstract

The characterization and conservation of materials constituting contemporary art pose a significant challenge for scientists and restorers due to the wide variability, heterogeneity in their compositions, and their chemical instability. This study aims to contribute to the understanding of the composition and ageing of materials in contemporary artworks, specifically, the analytical painting and the materials involved in a vandalic act. To this purpose, an acrylic painting on raw canvas is examined, *Tela* by Giorgio Griffa (1973), along with model samples purposely prepared together with the painter. Felt‐tip marker vandalic act, by which the painting was disfigured, is analyzed as well. The analytical approach entails a combination of noninvasive spectroscopic techniques to study the molecular composition and changes induced by artificial ageing in model samples. The results are correlated with those obtained on the painting to support the development of a suitable conservation treatment to remove the effects of vandalic act.

## Graphical abstract

Sequentially shifted excitation (SSE) Raman results on artificiallyaged acrylic‐based model paints and Tela (1973) canvas painting by Griffa suggest the surface enrichment in surfactant. Vandalic trait, with an orange felt‐tip pen, consists of an acrylic binder and azo‐dyes. Spectral markers, FTIR (1731 cm^−1^), and Raman (1600, 1502 cm^−1^), have been identified to support the monitoring of the vandalic trait removal.

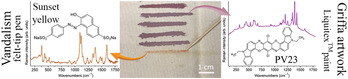

## 1 Introduction

Identifying the materials that constitute artworks is essential for developing effective conservation methods and resolving questions of authenticity. Synthetic polymers have been a common component in paint formulations for over 50 years, yet the development of effective analytical techniques for their identification in artworks has only recently evolved. It was not until the 1990s that pyrolysis–gas chromatography (Py–GC) was proven capable of reliably identifying most synthetic binders used in artists’ painting materials, especially when coupled to mass spectrometry (Py–GC/MS).^[^ ^1^ ^]^ Spectroscopic methods, such as Fourier transform infrared spectroscopy (FTIR), provide valuable, albeit limited, information about the composition of modern paints. However, their suitability for in situ measurements often makes them the preferred choice.

Modern paint binders are broadly classified in four principal polymer classes: acrylic, polyvinyl acetate (PVA), alkyd, and nitrocellulose.^[^ ^1^ ^]^ Acrylic has been the dominant binder in the artists’ paint market, commonly categorized as acrylic solutions (polymer is dissolved in a mineral spirit or turpentine) and dispersions (or emulsions) (polymer dispersed in water), developed respectively in the late 1940s and 1950s. In emulsion paints, acrylic and methacrylic copolymers have been employed in varying ratios to achieve the desired physical properties. The most commonly used copolymer was poly(ethyl acrylate/methyl methacrylate) (p[EA/MMA]), which, since the 1990s, has been replaced by poly(*n*‐butyl acrylate/methyl methacrylate) (p[nBA/MMA]) due to its superior hydrophobicity. Modified acrylic copolymers were also detected, such as styrene–acrylic and acrylic–vinyl, involving styrene and vinyl acetate, respectively.^[^ ^1^ ^]^ Besides the emulsion polymer binder, acrylic paints typically include pigments, water, emulsifiers (one or more surfactants), and various additives to enhance their performance, application, shelf life, and stability.^[^ ^1^ ^]^

Comprehensive knowledge of the specific polymeric binders, pigments, and additives used in original artworks or restoration materials is essential for conservators, as the chemical properties of these components influence the selection of treatment agents and methods. Additionally, understanding the composition and the evolution of paint formulations used by artists over time provides a valuable foundation for technical examinations, particularly in authenticity investigations.

**Figure 1:**
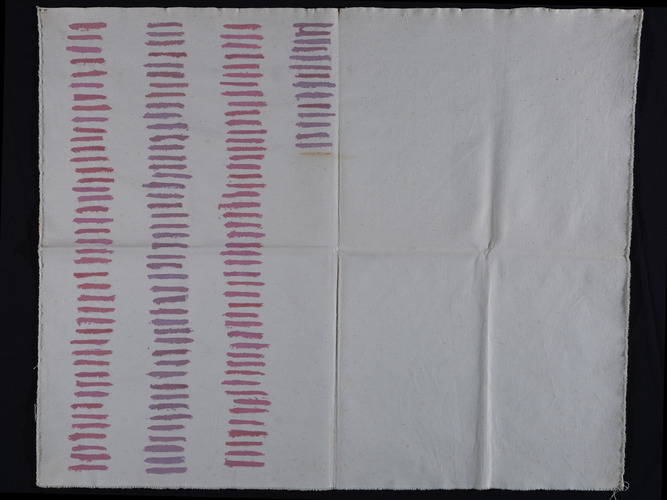
*Tela* painting by Giorgio Griffa (1973, Acrylic on raw canvas, 70.5 × 99.5 cm). Property of Museo della Città ‐ Polo Culturale Bottini dell'Olio ‐ Comune di Livorno. Photograph (2021, Roberto Bellucci (OPD), by permission of Ministry of Culture ‐ Opificio delle Pietre Dure.

The author of the painting examined in this study, Giorgio Griffa, focused in the 1970s on the analytical aspect of painting. Analytical painting (or Analytische Malerei), a term for the first time used by the critic Klaus Honnef in the catalog of the exhibition Geplante Malerei 17 in 1974–1975, developed in the early 1970s with the primary aim of conducting an internal analysis of the very nature of painting, through a process of reduction to its fundamental constituent elements.^[^ ^2^ ^]^ The painting *Tela* by Giorgio Griffa (**Figure** **1**), from the Museo della Città—Polo Culturale Bottini dell’Olio—Comune di Livorno (Italy), was analyzed to verify directly the information gathered through an interview with the painter, in preparation for a conservation intervention. The artwork was vandalized with a marker, probably in 1990. Therefore, the investigation aimed to identify both the constituent materials of *Tela* and those of the vandalism, with the specific requirement that the analyzes be completely noninvasive. To this end, model samples on canvas were created by the painter himself to assess the effectiveness of the analytical techniques and the instrumental response in a realistic scenario.

Valuable information about the artistic technique of analytical research can be drawn from programmatic texts, as well as from statements and interviews given by the artist. In this respect, the painter referred that in his paintings, he most likely employed Liquitex Heavy Body Acrylics, Violet Shade. As for the specific binder, Helwig and Poisson^[^ ^3^ ^]^ reported p(EA/MMA) in Liquitex paint samples from Yves Gaucher's studio (1934–2000), while Fardi et al.^[^ ^4^, ^5^ ^]^ revealed presence of Sty/nBA/MMA as the binder in Liquitex paints from 2014. The quantity of styrene is referred to as small and is revealed only by Py–GC/MS, not by attenuated total reflectance (ATR) FTIR. Ortiz‐Herrero et al.^[^ ^6^ ^]^ detected styrene (Sty) together with p(nBA/MMA) by Py–GC/MS in Liquitex paints purchased in 2015–2016, providing further evidence of changing formulation over time. As mentioned before, Py–GC/MS allows to identify components present even in low concentration, such as styrene component or surfactants, at the cost of sampling. Minor constituents may be difficult to be disclosed by FTIR analysis due to the detection limit.^[^ ^4^ ^]^ Several researchers also explored the stability of Liquitex paints, dealing mainly with the paint models cast on glass substrate^[^ ^5^, ^7^, ^8^ ^]^ or plastic sheets,^[^ ^6^ ^]^ relying on the possibility of sampling the material for the analytical investigation. However, as pointed out by Whitmore et al.^[^ ^9^ ^]^ for the paint films applied to canvas, the ageing process could have different routes, also due to the possible transfer of materials from the underlying canvas to the coating or to different paint morphology and distribution.

Felt‐tip pens experience an increased use in the realization of the modern artworks (sketches, drawings, copies, and architectural designs, etc.), and therefore, their chemistry has started to be investigated. Unfortunately, due to their esthetic effects, availability, and low costs, markers appear also in different vandalic acts. Generally, the felt‐tip pen inks are based on synthetic organic pigments (e.g., phthalocyanines, dioxazines, and azo pigments) and dyes (e.g., azo‐dyes, triarylmethanes, and xanthenes). Felt‐tip binders belong to families of carbohydrates (e.g., plant gums), styrene–acrylic resins (e.g., styrene methyl methacrylate in Faber‐Castell), polyamides, and polypropylene glycol resins. For their characterization, complementary analytical techniques may be exploited, such as thin‐layer chromatography (TLC); VIS‐reflectance spectroscopy; μ‐Raman, surface‐enhanced Raman, and FTIR spectroscopies; and mass spectrometries (e.g., GC/MS, Py–GC/MS and so on).^[^ ^10^, ^11^, ^12^ ^]^ While fundamental studies on reference pens might be performed using invasive methods, such as those based on mass spectrometry (MS), and for the in situ examination only noninvasive methods, such as Raman and/or FTIR spectroscopies (RS and FTIR), must be considered.

The aim of this research was to further the understanding of the materials used by Griffa and their behavior over time, with a view to fine‐tuning the cleaning methodology to remove the felt‐tip pen vandalism. To achieve this, a noninvasive analytical methodology was developed for investigating the artwork's creation process, chemical composition, and conservation state, including the composition of the vandalic act. For the first time, to the best of our knowledge, an approach was adopted in which the artist himself prepared model samples with paint layers on canvas supports, using materials and procedures consistent with those of the artwork. These samples contained Liquitex Heavy Body Acrylics (Violet Shade) alone or mixed with interior white paint (Sigma Coatings, Brandicolor Stumpfmatt line, Base LN). Such model samples, both in their natural state and after artificial ageing, were subjected to noninvasive and microinvasive analyzes, specifically ATR and portable reflectance (*r*) FTIR spectroscopy, dispersive micro‐Raman spectroscopy (RS) and portable sequentially shifted excitation RS (SSE RS), colorimetry, macro photographic documentation, and observation of polished cross‐sections under an optical microscope. Further, felt‐tip pen traits of various Italian brands (Sharpie, Giotto and Stabilo) were applied on the canvas to discern the chemical composition of the vandalic act on the artwork. The ageing behavior of the model samples was studied and correlated with data acquired from the artwork, providing critical insights to set up the most effective conservation methodology for the act of vandalism.

## 2 Results and Discussion

### 2.1 Model Painting Samples (Experimentation and Ageing)

**Figure 2:**
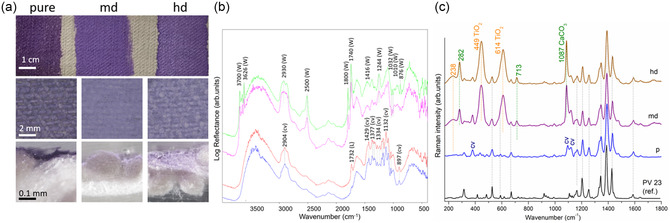
a) Model sample with Liquitex Dioxazione HB paint applied on canvas as: pure (p), medium diluition (md), and high diluition (hd), with white wall paint. Bottom row: optical microscopy observation of cross‐sections evidences greater penetration into the canvas fibers, woth increasing diluition. b) Reflectance FTIR spectra of cotton canvas (bottom‐up) unpainted (blue line) and painted with Liquitex Dioxazine pure (red line), and in mixture with wall paint at medium (pink line) and high diluition (green line). The main peaks of canvas (Cv) and Liquitex paint (L) are detected in pure paint, while white wall paint (W) is detected in md and hd paints. c) Portable SSE Raman spectra (bottom to top) of reference PV23 (KIK‐IRPA database), Liquitex pure, md, and hd. With high diluition the signals of calcite and TiO_2_, increase. The (micro)photographs of the canvas surface are provided by permission of Ministry of Culture ‐ Opificio delle Pietre Dure.

To study the materials used in the painting and their ageing behavior, model samples of paint layers (Liquitex Heavy Body (HB) Acrylics, USA) on textile support were prepared together with the painter G. Griffa. Liquitex HB Violet Shade paint was applied as pure or diluted with a white wall paint (SIGMA Coatings‐BrandiColor Stumpfmatt, color LN). A series of pure‐undiluted (p), medium dilution (md), and high dilution (hd) mock‐ups were, thus, created (**Figure** **2**a).

Characterization of the reference paint materials was first performed on films laid on glass slides by ATR‐FTIR spectroscopy. It disclosed the presence of *n*‐butylacrylate‐methymethacrylate p(nBA/MMA) copolymer in Liquitex paint, showing IR absorption bands at 2955, 2933, and 2874 (νCH); 1725 (νC=O); 1447 and 1385 (δCH); 1233, 1139, and 1066 (νC—O); and 983 (νC—C) cm^−1^ (Figure S1, Supporting Information).^[^ ^13^, ^14^ ^]^ The results testify the modification of the Liquitex acrylic paint formulation over years from p(EA/MMA) to p(nBA/MMA) in agreement with other studies.^[^ ^3^, ^4^, ^5^, ^6^ ^]^ In our study, the characteristic bands of styrene (=C—H aromatic stretching at 3100–2800 cm^−1^, C=C aromatic stretching at 1650–1450 cm^−1^, or C—H rocking of the aromatic ring at 761–702 cm^−1^) were not detected. This component, aimed at decreasing the Liquitex cost and improving the paint performance by augmenting its hardness and resistance to weathering, water, and alkali, is detectable, for example, by Py–GC–MS, and if present in low quantities, it might not be evidenced in FTIR spectrum.^[^ ^4^, ^5^ ^]^ Regarding other components, researchers reported a nonionic polyethoxylate (PEO) type surfactant in Liquitex paint, specifically octyl phenyl polyethoxy ethanol, with main absorption bands at 1114, 1341 and 962 cm^−1^.^[^ ^15^ ^]^ However, its concentration may be as low as 2%, and therefore, its signal may not be retrieved by FTIR spectroscopic methods, especially when performed without sampling, sample pretreatment, or separation,^[^ ^16^ ^]^ as in this study. In contrast, weak IR absorption bands (Figure S1a, Supporting Information) owed to the colorant are discernible at 1635, 1604, and 1572 cm^−1^ (C=C stretching vibrations in a conjugated aromatic system); 1553 cm^−1^ (C=C or C—N stretching); 1341 and 1116 cm^−1^ (C—C and C—O stretching); and 744–720 cm^−1^ (aromatic C—H out‐of‐plane bending), consistently with the literature.^[^ ^17^ ^]^ It is worth noting that the bands at 1341 and 1114 cm^−1^ detected in the Liquitex Dioxazine paint (Figure S1a, Supporting Information) are common to PV23 and PEO, and the band at 962 cm^−1^ overlaps with the acrylic, so, the presence of the surfactant cannot be excluded.

The interior white paint (Sigma Coatings brand, Brandicolor Stumpfmatt line, in the shade Base LN) (Figure S1b, Supporting Information) proved to be composed of kaolinite (characteristic FTIR absorption bands between 3700 and 3500 cm^−1^, corresponding to stretching frequencies of OH groups, Al—OH bending vibrations at 914 cm^−1^, Si—O—Al stretching at 542 cm^−1^, and Si—O—Si bending at 474 cm^−1^),^[^ ^18^, ^19^, ^20^ ^]^ calcite (fundamental vibrations from ${\text{CO}}_{3}^{2-}$ at 1416 and 876 cm^−1^ assigned to v_3_ and v_2_, respectively, and combination modes at 2500 and 1800 cm^−1^ assigned to v_1_ + v_3_ and v_1_ + v_4_, respectively),^[^ ^21^, ^22^ ^]^ and PVA (C—H stretching at 2930 cm^−1^, C=O stretching at 1740 cm^−1^, and C—(C=O)—C of ester groups at 1244 cm^−1^).^[^ ^23^ ^]^ When mixed with the Liquitex paint, the white wall paint dominates the ATR‐FTIR spectrum by masking the bands of the former (Figure S1b, Supporting Information).

Following this preliminary characterization, to verify the performance of the portable techniques and to simulate the measurements in situ, the model samples on canvas support (Liquitex paints pure, medium‐, and high‐dilution with white wall) were analyzed by reflection FTIR and by both bench and portable RS. Reflectance FTIR spectra revealed kaolin (3700–3626, 1032, and 1010 cm^−1^), calcite (2500, 1800, 1416, and 876 cm^−1^), and PVA binder (2930, 1740, and 1244 cm^−1^) of the wall paint in all the diluted samples. The IR bands of cotton canvas and Liquitex were revealed only in case of pure paint, where the acrylic polymer vibration (1732 cm^−1^) is clearly detected (Figure 2b). Nevertheless, the vibration of the acrylic polymer can be detected as a shoulder on the PVA band. The bands in the spectral region between 1500 and 897 cm^−1^ are ascribed to C—H, —OH, and C—O vibrations in cellulose.^[^ ^24^ ^]^ The slightly different intensity of the calcite vibration bands at 1800, 1416, and 876 cm^−1^ between medium and high dilution may reflect the different concentration of this component in the mixtures. It is noteworthy that the reflectance analysis does not detect the colorant in the case of pure paint, unlike what is observed in ATR. Furthermore, as already observed in ATR for the mixed paints, the only compounds that are also detected in reflectance are those present in the wall white (kaolin, calcite, vinyl polymer). In contrast, RS (Figure 2c) secures confident identification of the dioxazine colorant bands at all dilution options having important marker bands in the 1300–1600 cm^−1^ range. Indeed, the spectra display Raman lines consistent with that of violet pigment (PV) 23 as compared to the reference spectrum from KIK‐IRPA database.^[^ ^25^ ^]^ The main Raman lines of PV23 pigment, which belongs to the family of dioxazine heterocyclic compounds,^[^ ^26^ ^]^ are located at 1164, 1203, 1253, 1343, 1387, and 1427 cm^−1^. Additionally, it is possible to identify the presence of titanium white (TiO_2_)—in the tetragonal phase of titanium dioxide (rutile)—238 cm^−1^, 447 cm^−1^ symmetric stretching, and 613 cm^−1^ out‐of‐plane bending.^[^ ^27^ ^]^ The rutile Raman bands partially overlap with colorant bands in the low wavenumber region. The Raman spectra confirm presence of calcium carbonate (155, 283, 713, and 1087 cm^−1^)^[^ ^28^ ^]^ already detected by rFTIR. It is noteworthy that portable SSE RS also detects canvas signals^[^ ^29^ ^]^ (Figure 2c), whereas, micro‐RS reveals more superficial, paint‐related information (Figure S2, Supporting Information). This might be attributed to different sampling volumes of the two methods having the laser spots size of 100 × 500 μm and about 1 μm, respectively.

To complement the spectral information, the painted cotton samples prepared as cross‐sections were observed under the optical microscope (Figure 2a, bottom row). The paint penetration into the canvas augments with increasing dilution with white wall paint. The colourimetric measurements performed on the model samples evidence the paint dilution and consequent penetration by changes in L* (lightness) and b* (blue/yellow) parameters, while a* (red/green) parameter remains invariant. The measured values for p, md, and hd paints (L* = 48.2, 59.4, and 53.8 and b* = −23.2, −29.8, and −26.5) testify the increased paint penetration facilitated by the dilution.

**Figure 3:**
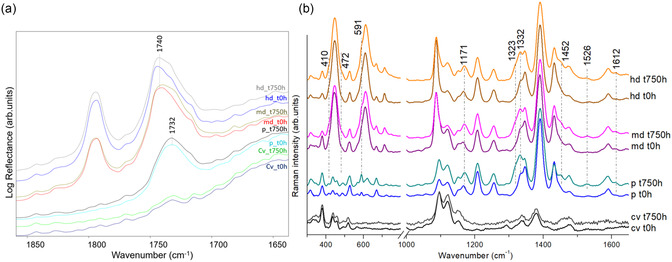
a) Reflectance FTIR spectra and b) portable SSE Raman spectra of the model samples: unpainted cotton canvas before (Cv_t0h) and after 750 h ageing (Cv_t750h); painted with pure Liquitex before (p_t0h) and after 750 h ageing (p_t750h); painteed with medium diluition mixture (Liquitex and white wall) before (md_t0h) and after 750 h ageing (md_t750h); and paint with high diluition mixture before (hd_t0h) and after 750 h ageing (hd_750h).

Regarding the paints’ behavior over time, reflectance FTIR analysis of the model samples before and after artificial ageing shows that the band attributable to the acrylic polymer in Liquitex (vC=O at 1732 cm^−1^), observed in the pure paint, does not change significantly as a result of 750 h ageing (**Figure** **3**a). In contrast, medium‐ and high dilution mixtures with wall white show a slight broadening of the vC=O band (1740 cm^−1^) of the vinyl polymer (Figure 3a). Ageing the acrylic paints has been investigated by various research groups.^[^ ^4^ ^]^ Mejía‐González et al. studied commercial acrylic emulsion paints and reported that the UV‐A radiation (400–315 nm, near‐UV) is usually considered harmless to the binder itself.^[^ ^8^ ^]^ Ormsby et al.^[^ ^30^ ^]^ demonstrated that after about 50 years of natural ageing of acrylic paintings, a substantial amount of surfactant may appear on the surface. The PEO‐based surfactant extracted from the Liquitex white paint was tentatively identified by FTIR as Triton X‐405.^[^ ^4^ ^]^ It was observed that being the glass transition temperature (*T*_g_) of acrylic emulsion polymers used in paints between 5–15 °C and PEO type surfactants’ melting temperatures between 40 and 50 °C, the variation of temperatures <15,40> °C might lead to exuding of the surfactant from the bulk of the paint toward its surface. The portable SSE Raman spectra (Figure 3b) of aged samples in this study (indoor ageing >310 nm for 750 h) could suggest the surfactant's migration toward the paint/air interface. The main spectral differences in aged samples (pure and diluted models) are in the presence of bands at 591 and 1452 cm^−1^ and the intensity increase of the 1171 and 1323–1332 cm^−1^ bands. The surfactant migration, studied by many research groups,^[^ ^6^, ^8^, ^15^, ^30^, ^31^, ^32^, ^33^, ^34^ ^]^ might be difficult to be discerned by reflectance FTIR due to the limitations, such as analytical detection thresholds (being surfactant about 2–6 wt%), and possible interference from substrate and bulk paint film materials, as in this case where the overlap of the main PEO band at 1341 cm^−1^ with that of the pigment makes attribution ambiguous.

The colourimetric measurements of the unaged and aged samples show significant increase of L* component (Δ*L* = 3.40, 2.16, and 1.90 contributing to the total color change (Δ*E*) of 5.07, 2.70, and 2.04 for pure, medium, and high diluted samples, respectively). The data suggest that the change is more evident in pure Liquitex paint samples. As reported by Digney‐Peer et al.^[^ ^31^ ^]^ PEOs can migrate and resurface as characteristic aggregates (crystalline or globular), affecting appearance of paint films.

### 2.2 Painting *Tela* by Griffa

The painting is composed by one hundred and forty‐six horizontal lines (Figure 1). The latter have a 5–6 cm length and a thickness ranging from 0.5 to 0.8 cm, which directly derives from the size of the brush chosen by Griffa. The lines are arranged on the canvas surface in four columns. The last horizontal line in the fourth and last column is orange and corresponds to the vandalic act and its characterization will be described in separate section.

**Figure 4:**
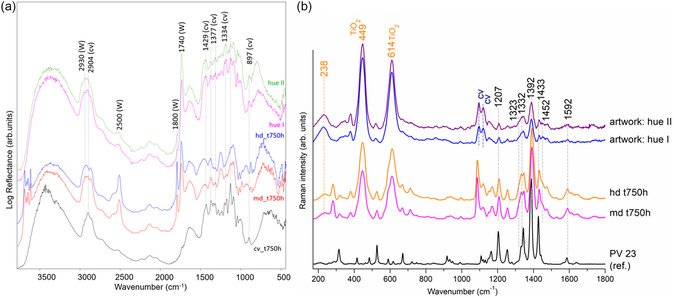
a) Reflectance FTIR spectra of *Tela* by Griffa on unpainted canvas after 750 h ageing (Cv_t750h); canvas painted with mixture Liquitex‐wall white at medium‐ and high‐dilution, after 750 h ageing (md_t750h and hd_t750h, respectively), as a reference; and hue I and hue II violet traits on the 4th column. The main peaks of canvas (Cv) and wall white paint (W) are labeled. b) Portable SSE Raman spectra of PV23 reference, canvas painted with Liquitex‐wall white md_t750h and hd_t750h, and artwork violet traits (hue I/hue II).

Reflectance FTIR spectra obtained on paint traits were compared with those collected on an unpainted area of the canvas and on the model canvas samples painted with the mixture of Liquitex and wall white at different dilutions (**Figure** **4**a). The presence of characteristic peaks of canvas (1429–897 cm^−1^) and of wall paint (mainly polymeric binder at 1740 cm^−1^ and calcite at 2500 and 1800 cm^−1^) suggests that the painter could have realized the investigated traits with a diluted mixture. Nevertheless, the typical bands of kaolin found in the medium‐ and high‐dilution models are not detected on *Tela*, suggesting that the white wall originally used did not contain this filler.

Afterwards, we focused on the characterization of the violet horizontal lines with RS. Thanks to the dimensions of the artwork, it was possible to analyze it both by bench RS coupled to microscope and by portable SSE RS. Several spectra were acquired across different tonalities, either warmer or cooler. Figure 4b shows the representative SSE RS acquired on two hues of violet traits. Micro‐RS spectra are reported in Figure S3, Supporting Information. The Raman spectra highlight the presence of the pigment PV23 (Dioxazine Violet pigment), along with the presence of titanium dioxide (TiO_2_) and calcium carbonate (CaCO_3_). This confirms the dilution with white wall paint as reproduced based on the author's suggestion in the model samples. From the TiO_2_/PV23 Raman lines ratios it can be deduced that the slightly different hues of the horizontal violet traits might be explained by varying ratio of Liquitex and white paint. The obtained spectra also suggest that the painter has never used undiluted Liquitex paint rather medium to high dilution ratio. This variability is probably to be attributed to the manual mixing of colors by the painter. Notably, the broadened 1323–1332 cm^−1^ bands and 1452 cm^−1^ are observed in SSE RS as in aged model samples.

### 2.3 Relating Model Samples with *Tela* Artwork by Griffa Through a Multivariate Data Analysis

**Figure 5:**
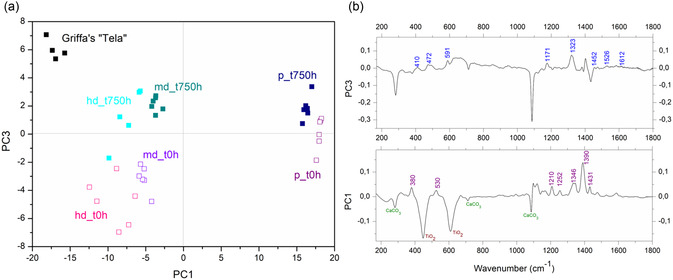
a) PC1/PC3 score plot reporting model samples Raman data with those retrieved on *Tela* painting (black rectangles). Model samples labeling ‐ p, md, and hd refer to pure, medium‐, and high diluted, respectively; t_0h (empty squares) and t_750h (full squares) refer to unaged and 750 h aged data, respectively. b) PC1 and PC3 loading plots.

As described earlier, the preliminary spectral examination revealed similarities between the model samples and the artwork, and shed some light on the painter technique. To perform an initial exploratory assessment of the Raman spectral dataset, a principal component analysis (PCA) was conducted to identify underlying patterns, trends, and potential outliers in the data. In this respect, the 170–1800 cm^−1^ spectral range was used to assemble the data matrix. After PCA computation, score maps were visualized (**Figure** **5**a) to identify the PCs yielding meaningful information (variance) to chemically characterize the model samples and relate them to the artwork data. The PC1/PC3 score plot, accounting for variance of 71.9% and 6.1%, respectively, allowed for a useful data separation, as a function of the paint dilution and degree of ageing, respectively. As shown in Figure 5a, several score clusters can be observed that are related to artwork, model samples (p, md, and hd)—unaged (t0h) and aged (t750h). The first principal component (PC1) accounts for the main separation between samples enriched in pure PV23 pigment (p specimen, dark, and purple squares) and those containing white materials also (md and hd). Aged samples are predominantly associated with positive PC3 values. In this respect, Raman data obtained from *Tela* painting (black rectangles) are clustered in the region with negative PC1 and positive PC3 values, collocating them with aged and highly diluted samples. The higher PC3 scores for the artwork data may be possibly explained by the fact that the model samples simulate roughly 15 years of natural ageing,^[^ ^6^ ^]^ while the artwork has been created in 1973 (natural age about 50 years in the time of experiments).

The vibrations mainly responsible for sample clustering can be observed by inspection of the loading plots for each variable for the first and third principal components (Figure 5b). For positive (dark blue and purple squares) and negative (all other squares) score values along PC1, the loadings reveal that a crucial role in describing the group of samples located at high score values along PC1 is played by well‐defined bands at 380, 530, and 1390 cm^−1^ related to the PV23 pigment. In contrast, negative score values along PC1 are influenced by the presence of 280 and 1089 cm^−1^ (calcite) and 449 and 614 cm^−1^ (TiO_2_) bands. On the contrary, aged and unaged samples are described by positive and negative score values, respectively, along PC3. The corresponding loading plot reveals the difference in aged samples (positive score values) in the presence of bands at 591 cm^−1^ (as a shoulder of TiO_2_, 614 cm^−1^) and 1452 cm^−1^, and intensity increase and broadening of the 1171 and 1323 cm^−1^ bands. Spectral features with negative PC3 loadings (e.g., calcite) indicate inverse contributions to the PC3 component, likely arising from their covariance with other variables in the dataset.

### 2.4 Vandalic Act on *Tela* by Griffa

**Figure 6:**
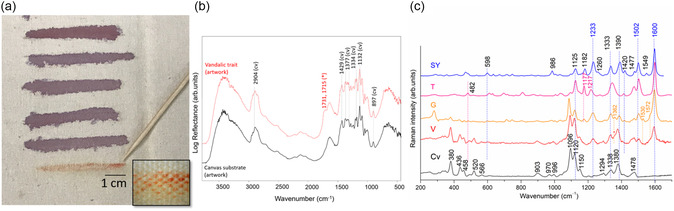
a) Griffa's artwork ‐ detail of orange vandalic trait and b) portable reflectance FTIR spectra acquired on canvas substrate (black line) and on vandalic trait (red line). Presence of 1731–1715 cm^−1^ band, attributable to C=O stretching, is indicative of the vandalic trait binder, c) portable SSE Raman spectra (bottom to top) of the canvas (cv) substrate (black line), vandalic (V) trait (red line), and orange marker brand Giotto (G) applied on canvas (orange line), reference of Tartrazine (T) and Sunset Yellow (SY) (pink and blue lines, respectively).

The vandalic act consisted of a linear element located in the last row of the fourth column (**Figure** **6**a). Positioned in the center of the artwork, beneath the last violet line, an orange line appeared to be made with a felt‐tip marker pen. The line is similar in shape and size to the artist's original brushstrokes, but distinguishable from the overall color scheme of the artwork under analysis. Through an interview with the artist, it has been confirmed that the orange line is foreign to the artist's work and its removal is desirable for the correct reading of the artwork. Identifying its chemical composition was therefore crucial for the definition of the suitable cleaning methodology for the controlled and optimal removal of the mark. Knowledge of the exact composition is also an essential step in understanding chemical behavior over time and fine‐tuning the conservation treatments.

The available literature reports on four different classes of binders revealed with Py–GC/MS: 1) acrylic emulsions, 2) ketone resins, 3) phenolic resins, and 4) benzene/naphthalene derivates.^[^ ^12^ ^]^ While portable reflectance FTIR spectroscopy has reduced sensitivity to the minor components, it proved useful in revealing information regarding the binder type of the felt‐tip pen involved in the vandalic act. In fact, the FTIR band at 1731/1715 cm^−1^ can be attributed to the C=O functional group contained in ester‐type binders, such as the acrylic ones (Figure 6b).

The literature and spectral references referred to felt‐tip pen markers are not extensive.^[^ ^10^, ^11^, ^12^ ^]^ As reported, orange pen inks are usually a mixture of red and yellow dyes, which may vary over different production periods.^[^ ^11^ ^]^ To prevent the identification of only major component, the analytical protocol involves typically their separation through TLC followed by spectral analysis. In this regard, RS has proven particularly suitable due to its sensitivity to coloring agents. In our study, the analytical campaign was restricted to exclusively noninvasive methods. The first attempt to identify the dye composing the vandalic mark on artwork with traditional micro‐Raman analysis failed due to high fluorescence background. In contrast, the portable SSE RS provided some meaningful results as follows.

Functional references, a series of orange marker brands commercially available on the Italian market for identifying the chemical composition of the marker used in the vandalic trait, were created. Brands, such as Sharpie, Giotto, and Stabilo, proved relevant to our study. For each marker, lines were made on a canvas with similar characteristics to that composing the artwork. The most prominent spectral similarities with the vandalic trait were observed for the Giotto and Stabilo markers (Figure S4, Supporting Information). The preliminary investigation pointed to ‐azo‐based dyes composing the vandalic trait. Therefore, we focused on further refining this aspect.

Since cellulose is a good Raman scatterer, dye identification is influenced by signals from the canvas as can be seen in the spectrum acquired on the artwork's canvas substrate (Figure 6c), with bands at 380, 436, 458, 520, and 566 (CCC, CO, CCO, cellulosic ring deformation); 903 (CH_2_ stretching); 1096 and 1120 (COC stretching symmetric); 1338 (HCH wagging bending and HCC, HOC, and COH rocking bending); 1380 (CH_2_ and C—C cellulosic ring asymmetric stretching); and 1478 cm^−1^ (HCH scissoring bending).^[^ ^29^ ^]^ The spectrum of vandalic trait (Figure 6c) is also dominated with the cellulose Raman lines but for the signals at 1600, 1502, 1362 (shoulder), and 1233 cm^−1^. These Raman signals match with those obtained from orange Giotto marker (Figure 6c). Sodo et al.^[^ ^11^ ^]^ identified, by separation (TLC) followed by RS, three dyes: 1) orange Sunset yellow (E110), 2) yellow Tartrazine (E102), and 3) red Ponceau4R (E124) in Giotto marker. The reference spectra (available from KIK‐IRPA spectral database) of the first two dyes are plotted for comparison in Figure 6c. The observed lines 1600, 1502, and 1233 cm^−1^ in Giotto and vandalic act traits can be attributed to the orange Sunset Yellow (E110) azo‐dye. The most prominent peak at 1600 cm^−1^ corresponds to the in‐plane bending of OH, the asymmetric stretching of COO^−^, the combined effect of symmetric stretching, and the out‐of‐plane C—H deformation of the two phenyl rings. Another major peak at 1502 cm^−1^ may be associated with phenyl ring in‐plane bending. The peak about 1233 cm^−1^ can be assigned to δNH, νN—N, and νCC. For tartrazine, the latter is observed at slightly lower wavenumbers (1217 cm^−1^).^[^ ^35^ ^]^ In both the vandalic trait and Giotto marker, the detected band is located at 1233 cm^−1^, suggesting that sunset yellow is dominating the spectrum. The presence of red dye, Ponceau4R (E124, Cochineal Red, New Coccine), could be deduced only by the shoulder at about 1572 cm^−1^ and a weak band at 1362 cm^−1^.^[^ ^36^ ^]^ This monoazo dye, containing two aromatic rings connected through azo bond (Ar—N=N—Ar) with ortho hydroxyl group, provides the possibility of tautomerism (‐azo and ‐hydrazo forms).^[^ ^37^ ^]^ Being RS sensitive to both forms, the main spectral bands are observed at 1622 and 1588 cm^−1^ in the ‐azo isomer, while for the —NH isomer at 1574, 1515, and 1365 cm^−1^. The latter bands can be observed in the Giotto marker suggesting the prevalent spectral contribution of —NH isomer of Ponceau4R, and in the vandalic trait.In contrast, the Sharpie marker seems to contain predominantly the —OH isomer of Ponceau4R with bands located at 1622, 1588, 1462(sh), and 1422 cm^−1^ (Figure S4, Supporting Information).

From the results of the Raman spectral investigation, it follows that the bands at 1233, 1502, and 1600 cm^−1^, attributable to the presence of the orange Sunset Yellow (E110) dye, can be exploited as the markers to monitor the efficacy of the cleaning process. Especially bands 1600 and 1502 cm^−1^ do not interfere with Raman lines of the substrate. A comparison between reflectance FTIR spectra collected on the vandalic trait and those obtained from felt‐tip marker references (Figure S5, Supporting Information) previously analyzed with Raman may further support the use of a marker containing acrylic binder. In fact, albeit weak, vibrations at 1731 and 1715 cm^−1^ are detected, consistent with this type of binder. In contrast, the Stabilo felt‐tip markers contain mostly carbohydrate binder, with some exception of (styrene)–acrylic resin.^[^ ^10^ ^]^ Not interfering with the spectrum of cellulose, the 1731/1715 cm^−1^ bands may be used as a marker to monitor the vandalic trait removal from the canvas substrate.

## 3 Conclusion

In this research, we examine the behavior of acrylic‐based model paints (Liquitex Heavy Body) in reference to the modern painting by Giorgio Griffa‐*Tela* (1973). The model samples were prepared in collaboration with the painter to simulate and explore the materials used in the painting and to develop a noninvasive analytical protocol for their examination. While numerous studies have addressed the ageing of acrylic paints, this is, to the best of our knowledge, the first study in which with paints were applied directly on canvas substrate to reproduce the real case scenario, by the artist himself. The main reason for using a canvas substrate was to test performance of the analytical methods and be able to correlate such data with those obtained on artwork. Artificial ageing was set to simulate indoor conditions (>310 nm) for 750 h, corresponding to ≈15 years of natural ageing. The noninvasive analytical protocol, exploiting portable FTIR and Raman spectral methods, enabled complementary characterization of both the model samples, the artwork, and the associated vandalic act. The analysis confirmed the use of Liquitex paint containing dioxazine violet (PV23), diluted with PVA‐based white wall paint, as previously indicated in the artist's interview. This interdisciplinary study also enabled the correction to be made in the museum catalog, where the artwork had originally been listed as an oil painting on raw canvas. Infrared data showed that white wall paint formulation changed over time. It originally contained rutile and calcite (as inferred by Raman and FTIR) while the new formulation (2020) includes kaolin also.

With respect to ageing, the detected variations in the infrared spectra mainly reflect changes in the vinyl polymer used as a binder in the white wall paint. Although this technique did not detect surface enrichment with the PEO‐based surfactant, probably due to spectral complexity, SSE Raman data suggest that such enrichment may have occurred. The trends observed in the Raman spectra of the artificially aged model samples correlate well with those observed in the painting.

Regarding the vandalic act, portable reflectance FTIR spectroscopy identified binder type, while to detect the composition of different organic dyes, the portable SSE RS, optimized for the removal of fluorescence background, proved fundamental. By comparing reference spectra, it was disclosed that the orange vandalic trait has been inferred with a felt‐tip pen composed by azo‐dyes (such as orange Sunset Yellow, yellow Tartrazine, and red Ponceau 4R), probably bound with an acrylic binder. By comparing the Raman spectrum acquired from the vandalic trait with those of the orange strokes of various markers, it was possible to find the most similarities with the Giotto brand. Spectral markers were identified, FTIR at 1731/1715 cm^−1^ for the binder and the Raman at 1600 and 1502 cm^−1^ for the dyes. These markers can be exploited in noninvasive analytical monitoring to support conservation efforts aimed at removing the vandalic trait from the canvas support.

## 4 Experimental Section

4.1

4.1.1

##### Model Sample Preparation

The model samples used in this study were created together with Giorgio Griffa, using materials available and used in his studio in 2020. Specifically, cotton canvas was used as a support. The fabric was used as it is, without washing to remove the industrial sizing or undergoing the humidification and tensioning treatment. Three types of paint mixtures (pure, medium dilution, and high dilution) were prepared in duplicate and applied with a 2 cm‐wide brush on canvas, as follows. Two pure (p) samples were prepared using Liquitex Heavy Body Acrylics ‐ Dioxazine Purple (lightfastness II, series 2), mixed with small amount of water (1 g of paint with 2.5 mL of water). Four medium and high dilution samples were prepared by mixing Dioxazine Purple with an interior white wall paint from Sigma Coatings, Brandicolor Stumpfmatt line, in the Base LN tone, in a ratio of ≈1:6. These four samples differ in the amount of water added, specifically, one pair labeled as medium dilution (md) and the other as high dilution (hd), were mixed with 9 and 27 mL of water, respectively.

##### FTIR and Raman Spectroscopies

FTIR spectra were collected with a portable Bruker Optics ALPHA FTIR Spectrometer equipped with a SiC Globar source and a DTGS detector. Reference paints laid on glass slides were scraped and analyzed using a Platinum ATR single reflection diamond module collecting 24 scans, in the 4000–400 cm^−1^ spectral range, with a resolution of 4 cm^−1^. Both the model samples and the artwork were characterized noninvasively using an External Reflection module, with a 6 mm measurement area diameter and background on a gold mirror. Spectra were recorded directly on the object surfaces in the range of 7000–375 cm^−1^, collecting 128 scans with resolution 4 cm^−1^. The spectra were processed using OPUS 7.2 software (Bruker Optics GmbH, Ettlingen, Germany).

Micro‐RS was performed with a benchtop micro‐Raman instrumentation Renishaw InVia equipped with an optical microscope (Leica DM2700), two solid‐state laser sources with wavelengths of 532 and 785 nm (spectral resolution 3 cm^−1^), and a thermoelectrically cooled CCD detector. The acquisition parameters used for the analyzes were as follows: a 785 nm laser with power maintained below 4 mW and an exposure time of 10 s with 5–10 accumulations. The calculated spot size for the 50× objective used in the analysis of the samples was 0.95 μm.

Portable SSE Raman spectrometry was performed with a BRAVO instrument (Bruker) equipped with two temperature‐controlled diode lasers (DuoLaser) at 785 and 852 nm, detected with different areas of the charged‐coupled device (CCD) with a spatial resolution in the range of 10–12 cm^−1^. Spectra could be acquired over an extended spectral range in two sequential steps: from 170 to 2200 cm^−1^ using the 852 nm laser and from 1200 to 3200 cm^−1^ using the 785 nm laser. Each laser was shifted three times based on a small temperature variation, resulting in three raw spectra shifted by ≈6 cm^−1^ for each laser, totaling six raw spectra. The algorithm processes these to produce a final SSE spectrum over the entire spectral range (170–3200 cm^−1^), free from fluorescence and other background interference. The acquisition parameters used for the analyzed samples were as follows: an exposure time of 500 ms and 50 accumulations. The laser spot on sample was about 100 × 400 μm.

##### Optical Microscopy and Colorimetry

Zeiss Axioplan optical microscope with visible and ultraviolet light; Leica stereomicroscope, model M250C; and Konica‐Minolta CM‐2600d colorimeter were utilized for the optical observation and colourimetric measurements. The L*a*b* coordinates (D65 illuminant, the observer at 2°) were used to calculate the color change Δ*E* = (ΔL*^2^ + Δa*^2^ + Δb*^2^)^−1/2^, where ΔL* value describes a change in brightness and its value ranges between 100 (white) and 0 (black). The a* and b* values represent the color directions: +a* is red and −a* is green, and +b* is yellow and −b* is blue, ranging between +60 and−60.

##### Artificial Ageing

After initial characterization (t_0h), the model samples were artificially aged for 360 h (t_360h) and 750 h (t_750h) in a solar box. The artificial ageing was performed in solar box (CO.FE.ME.GRA. 3000e model) equipped with a Xenon lamp with a nominal irradiance of 500 W m^2^, filtered for wavelengths below 310 nm (simulating indoor conditions), and a black panel temperature of 60 °C, following ISO 11341/2004 (Paints and varnishes ‐ Artificial weathering and exposure to artificial radiation ‐ Exposure to filtered xenon‐arc radiation). The accelerated ageing process was designed to simulate ≈15 years of natural ageing.

##### PCA

PCA analysis was applied for exploratory purposes to Raman datasets, using the range 170–1800 cm^−1^. Suitable row pretreatments were selected and applied in attempt to minimize systematic unwanted variations. In more details, the standard normal variate transform is a pretreatment used for both baseline shifts and global intensity variations in the spectral data. After the preliminary map analysis, score, and loading plots were examined to identify the spectral bands that mainly describe each sample and better evaluate the information. The spectral range considered from the obtained spectra was analyzed with PCA using the Chemometric Agile Tool software.

## Conflict of Interest

The authors declare no conflict of interest.

## Supporting information

Supplementary Material

## Data Availability

The data that support the findings of this study are available from the corresponding author upon reasonable request.
