# Supplementary material for: Spectroscopic Investigations of a Vandalized Contemporary Acrylic Painting on Canvas Using Model Paintings and Chemometrics
Source: Chempluschem. 2025 Jun 13;90(8):e202500129. doi: 10.1002/cplu.202500129 (PMC12352722; doi:10.1002/cplu.202500129)
Supplement: Supplementary file 1 — Supplementary Material [file CPLU-90-e202500129-s001.pdf]

ChemPlusChem

Supporting Information

# Spectroscopic Investigations of a Vandalized Contemporary Acrylic Painting on Canvas Using Model Paintings and Chemometrics

Jana Striova,* Silvia Innocenti, Arianna Ingrassia, Moira Bertasa, and Barbara Salvadori

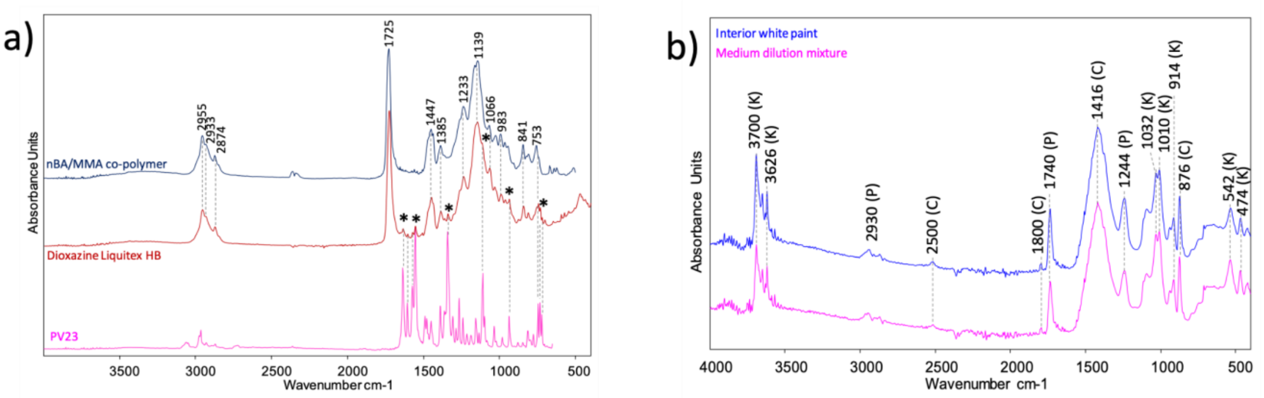


**Figure S1.** ATR FTIR spectra acquired on materials applied on glass slides: (a) top-reference nBA/MMA co-polymer (IRUG database) middle- Dioxazine Liquitex HB, bottom-reference PV23 (IRUG database). The asterisks mark the bands of the colorant. (b) top-the interior white paint showing the main peaks of kaolinite (K), calcite (C) and polyvinylacetate (P) and bottom- a mixture of Liquitex paint with white paint (medium dilution).

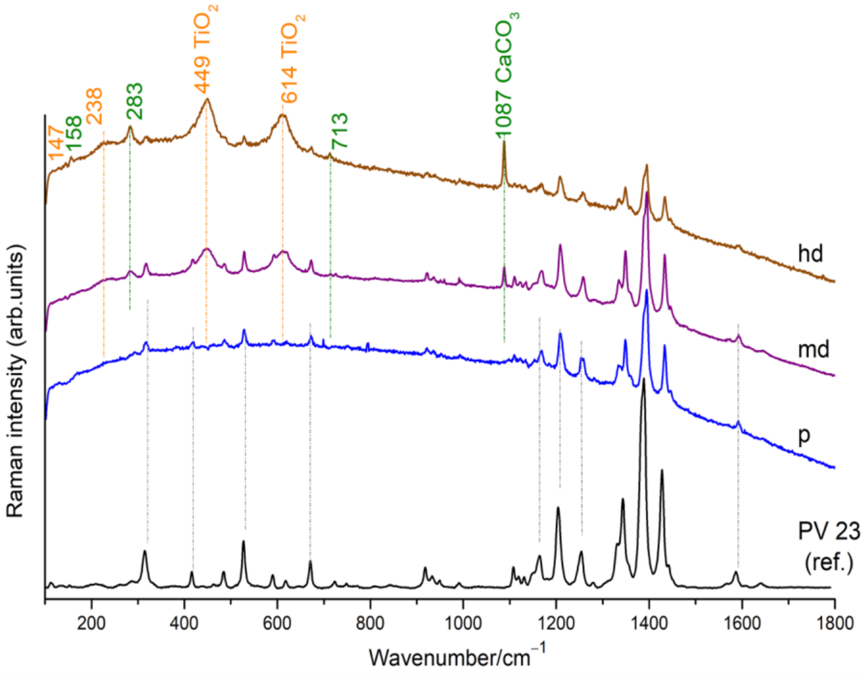


**Figure S2.** Micro-Raman spectra (bottom to top) of reference PV23 (KIK-IRPA database), Liquitex pure, md and hd paints. With high dilution the signals of calcite and $TiO_2$ increase. Signals of canvas are not detected.

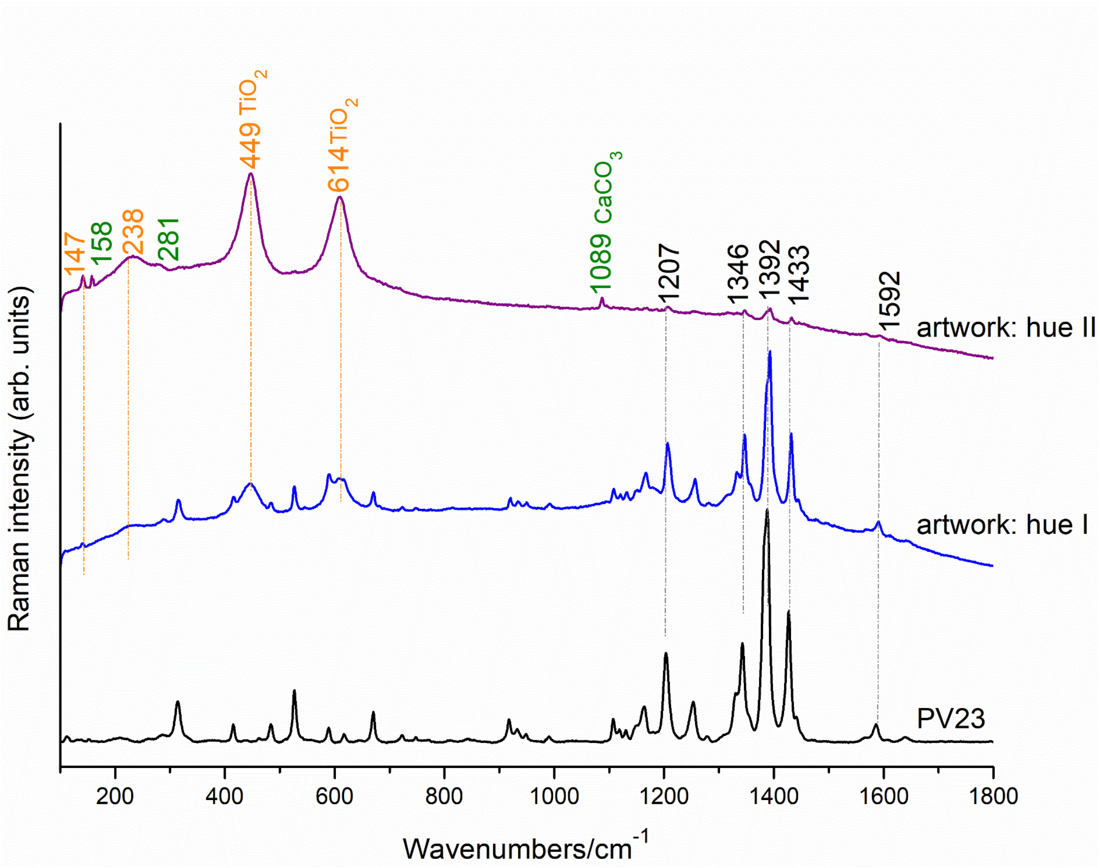


**Figure S3.** Micro-Raman spectra: bottom PV23 reference and top: artwork violet traits (hue I/hue II).

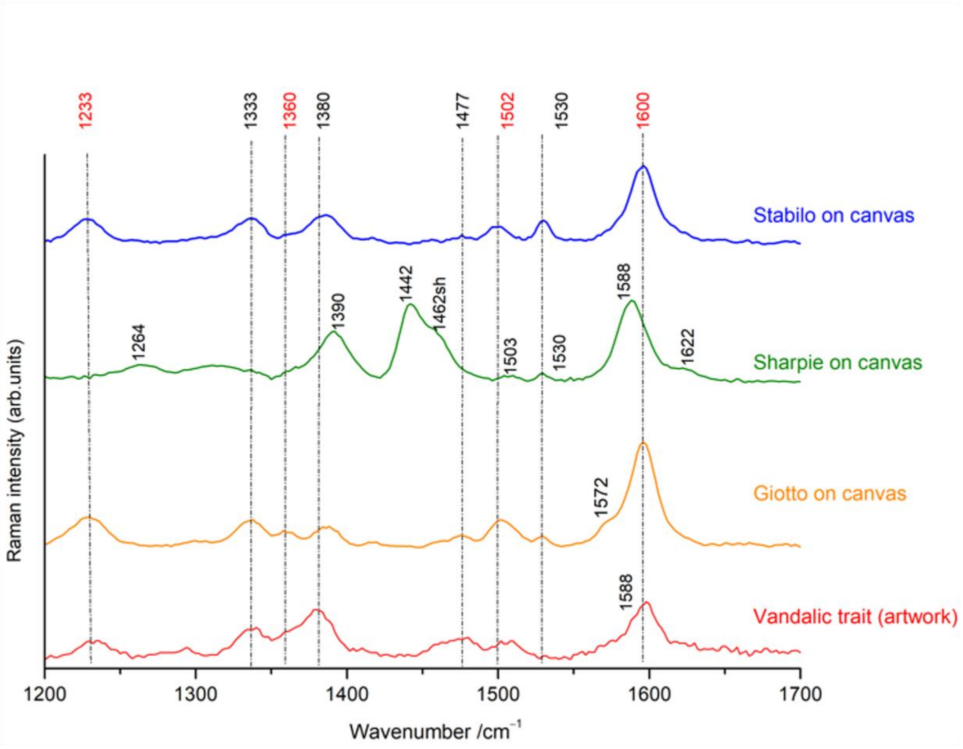


**Figure S4.** Portable SSE™ Raman spectra (bottom to top) of the vandalic trait (red line), markers on canvas: Giotto (orange line), Sharpie (green) and Stabilo (blue).

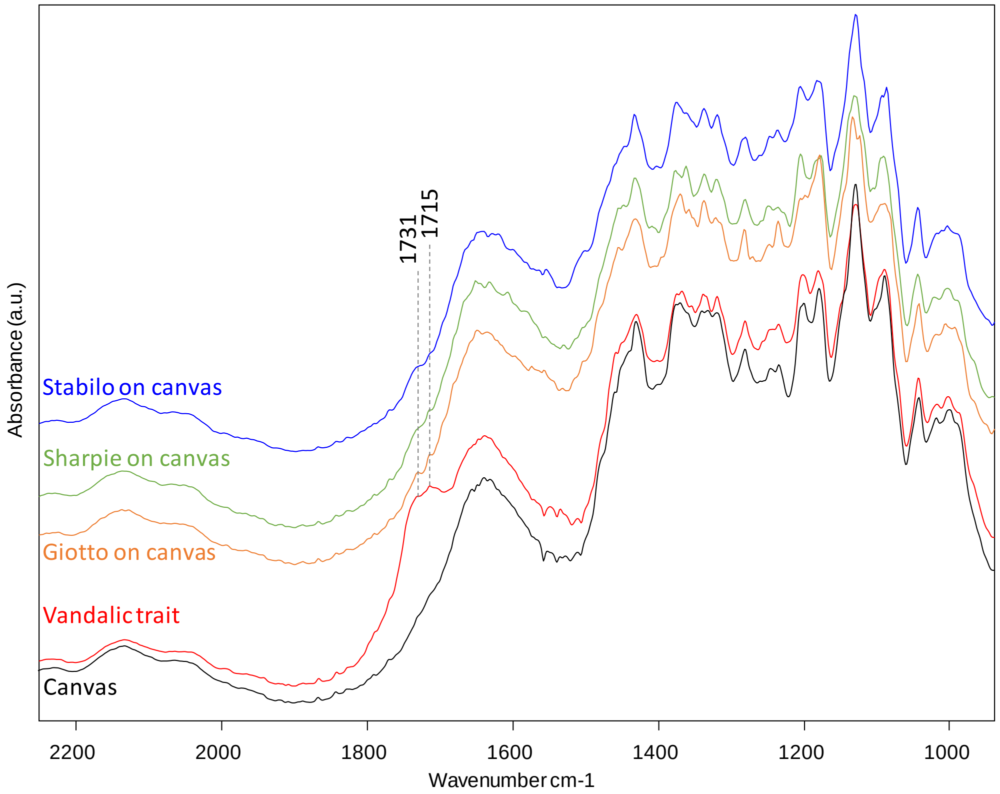


**Figure S5.** Portable reflectance FTIR spectra acquired on canvas substrate (black line) and on vandalic trait (red line). Markers on canvas: Giotto (orange line), Sharpie (green) and Stabilo (blue).
